# Large-Scale Functional Networks, Cognition and Brain Structures Supporting Social Cognition and Theory of Mind Performance in Prodromal to Mild Alzheimer’s Disease

**DOI:** 10.3389/fnagi.2021.766703

**Published:** 2021-11-17

**Authors:** Jose Manuel Valera-Bermejo, Matteo De Marco, Micaela Mitolo, Chiara Cerami, Alessandra Dodich, Annalena Venneri

**Affiliations:** ^1^Department of Neuroscience, University of Sheffield, Sheffield, United Kingdom; ^2^Department of Life Sciences, Brunel University London, London, United Kingdom; ^3^Functional and Molecular Neuroimaging Unit, IRCCS Istituto delle Scienze Neurologiche di Bologna, Bologna, Italy; ^4^IUSS Cognitive Neuroscience (ICoN) Center, University School for Advanced Studies IUSS-Pavia, Pavia, Italy; ^5^Cognitive Computational Neuroscience Research Unit, Mondino Foundation IRCCS, Pavia, Italy; ^6^Center for Mind/Brain Sciences (CIMeC), Università degli Studi di Trento, Rovereto, Italy

**Keywords:** Alzheimer’s disease, social cognition, theory of mind, fMRI, VBM, mild cognitive impairment

## Abstract

Impairment of social cognition (SC) skills such as recognition and attribution of intentions and affective states of others (Theory of Mind, ToM) has been evidenced in Alzheimer’s Disease (AD). This study investigated the neuropsychological, neuroanatomical and brain-functional underpinnings of SC processing to obtain an understanding of the social neurophenotype in early probable AD. Forty-six patients with mild cognitive impairment and mild probable AD underwent SC assessment including emotion recognition (Ekman-60-faces task) and cognitive and affective ToM (Reading-the-Mind-in-the-Eyes test and Story-based Empathy task). Linear models tested the association between SC scores and neuropsychological measures, grey matter maps and large-scale functional networks activity. The executive domain had the most predominant association with SC scores in the cognitive profile. Grey matter volume of the anterior cingulate, orbitofrontal, temporoparietal junction (TPJ), superior temporal, and cerebellar cortices were associated with ToM. Social cognition scores were associated with lower connectivity of the default-mode network with the prefrontal cortex. The right fronto-parietal network displayed higher inter-network connectivity in the right TPJ and insula while the salience network showed lower inter-network connectivity with the left TPJ and insula. Connectivity coupling alterations of executive-attentional networks may support default mode social-cognitive-associated decline through the recruitment of frontal executive mechanisms.

## Introduction

Theory of Mind (ToM) is defined as self-originating inferences about other people’s intentions, beliefs and emotions that guide decision making and modulate behaviour in accordance with established social standards (Baron-Cohen et al., 1985). ToM can be further divided into cognitive ToM (attribution of intentions/beliefs) and affective ToM (emotional states) that are said to be controlled by partly independent neural systems (Kalbe et al., 2010; Abu-Akel and Shamay-Tsoory, 2011). Moreover, a second affective component of ToM, known as “cognitive empathy” (Dvash and Shamay-Tsoory, 2014), introduced as an element of connection between the former two, would be responsible for successful emotion recognition at the basis of affective mental state attributions (Mier et al., 2010; Mitchell and Phillips, 2015).

Impairment of ToM has been evidenced in Alzheimer’s disease (AD) (Freedman et al., 2013; Moreau et al., 2016; Chainay and Gaubert, 2020; Kessels et al., 2021), and may occur early, even at the prodromal stage of disease (Bora and Yener, 2017; Yildirim et al., 2020). However, this deterioration is not as severe as that observed in other neurocognitive domains such as for example in memory (Dodich et al., 2016). Furthermore, AD displays significantly less severe social cognition deficits compared with other forms of neurodegeneration, such as Lewy body dementia (Heitz et al., 2016), or the behavioural variant of fronto-temporal dementia (Bora et al., 2015). Therefore, social cognition abilities have been proposed as useful cognitive markers for discriminating among different forms of dementia (Bertoux et al., 2016; Dodich et al., 2018).

The most prevalent behavioural framework posits that detriments of ToM and social cognition in AD are a by-product of cognitive dysfunction, particularly in the executive domain (Dodich et al., 2016; Ramanan et al., 2017; Christidi et al., 2018; Torres et al., 2019). Based on this outline, social cognition impairment would initially hinder complex functions that rely heavily on attention, reasoning and decision-making (i.e., detection of second-order false beliefs), compromising more basic social functions (i.e., recognition of basic emotions) at later stages (Castelli et al., 2011; García-Rodríguez et al., 2012). Furthermore, the neural substrates sustaining ToM and social cognition in AD are still poorly understood and differ across cognitive or affective social skills (Poletti et al., 2012). Research carried out in the general population has identified a candidate neural network associated with mentalising skills that encompasses the medial prefrontal cortex (mPFC), temporoparietal junction (TPJ), posterior superior temporal sulcus and precuneus (Gallagher and Frith, 2003; Saxe and Wexler, 2005; Frith and Frith, 2006; Schurz et al., 2014). Some of these regions have also been found actively involved in supporting executive functions in the prodromal to mild AD continuum (Habeck et al., 2012). Moreover, most of these regions contribute to a large-scale system known as default-mode network (DMN) (Schilbach et al., 2008; Li et al., 2014). Although the DMN is a “task-negative” network that deactivates during task engagement, its computational hubs also contribute to “task-positive” activation patterns in support of executive control (Wade et al., 2018).

Early dysfunction of the DMN is a distinguishable pathophysiological hallmark of early stage AD, even at the prodromal Mild Cognitive Impairment (MCI) stage (Greicius et al., 2004; Zhang et al., 2012; Badhwar et al., 2017). As a result, additional executive/attentional resources might be required to demonstrate an adequate social cognitive performance in the presence of a down-regulated DMN. Limited neuroimaging studies have investigated the neural substrate of ToM abilities in the prodromal MCI and mild AD clinical phases, and these studies have mainly focussed on cognitive ToM. A first task-based fMRI study found reduced activation of fronto-temporal and subcortical regions in amnestic MCI patients during false belief tasks (Baglio et al., 2012). A second research study explored volumetric indices of brain structure in AD and found a significant association between mentalising skills and volume of hippocampal and cerebellar regions (Synn et al., 2018). A third study focussed on regional metabolic patterns found an association between the left TPJ and ToM false-belief reasoning in mild AD patients (Le Bouc et al., 2012). Lastly, a SPECT perfusion study found an association between ToM (false belief) and blood flow in the posterior cingulate (Takenoshita et al., 2020). Although these studies have provided useful insights on the possible neural underpinnings of ToM deficits in AD, their considerable level of heterogeneity indicates that the overall emerging pattern is still inconclusive.

The present study aimed thus to test the association between ToM and associated social cognition abilities and behavioural, structural and functional outcomes in order to provide an integrative social brain/neurocognitive profile across the prodromal to mild AD continuum and expand the limited data available in the current literature on this population. To our knowledge, this is the first comprehensive study to investigate domain-specific cognitive and affective ToM correlates in early-AD individuals through multi-task social cognition assessment and multi-modality neuroimaging acquisition.

In the context of the above-mentioned findings on normal individuals and AD patients, we hypothesised that ToM abilities in early-AD would be associated with volumetric integrity of fronto-parietal and limbic structures that represent the territory where the DMN is expressed. We also hypothesised that social cognition and complex ToM performance, at a functional level, would be supported by executive and attentional networks (and associated cognitive functions) in the presence of a vulnerable DMN.

## Materials and Methods

### Participants

Forty-six patients were recruited for this study from our outpatient memory clinic neuropsychology services. All participants included in this study met clinical criteria for a diagnosis of MCI (*n* = 37, MMSE range 24–30) (Petersen, 2004; Albert et al., 2011) or mild stage probable AD dementia (*n* = 9, MMSE range 21–23), following the National Institute on Ageing criteria (McKhann et al., 2011; Jack et al., 2018). Initial diagnosis was confirmed for all patients through comprehensive longitudinal clinical neurological and neuropsychological assessment with support of structural magnetic resonance imaging (MRI) scans and clinical monitoring occurring over a period of at least four years.

A sample of 34 healthy controls (16 males and 18 females) matched for demographic characteristics and who did not meet any of the exclusion criteria set for the study was also included for comparison of patients’ neuropsychological and neuroimaging profiles. This sample of healthy participants was not involved in the social cognition experiments and was included for the sole purpose of demonstrating that the patterns of cognitive deficits and neuroimaging abnormalities of patients were typical of early AD.

Main exclusion criteria for all study participants were set as follows, and were used to rule out the interfering effects of other types of neurological conditions: presence of cognitive fluctuations or neuropsychiatric symptoms compatible with types of neurodegenerative dementia diagnosis other than Alzheimer’s disease, acute/chronic cerebrovascular disease or history of transient ischaemic attacks, uncontrolled brain seizures or history of epilepsy, peripheral neuropathy disorders, neuropsychiatric or other neural conditions not compatible with our study as detected by MRI, cardiovascular and gastroenterological conditions such as sick-sinus syndrome or peptic ulcer, metabolic disorders such as abnormal levels of B12, folates or thyroid-stimulating hormone, pharmacological interventions such as pre-recruitment treatment with memantine/cholinesterase inhibitors, psychotropic medication, pharmacological components displaying important organic adverse effects or medications used in other research protocols and presence of major disabilities that could impact negatively on cognitive or everyday life functions. All participants undergoing chronic treatment for other severe non-neurological diseases were on stable dosage during data acquisition.

Ethical approval was granted by the Regional Ethics Committee (Protocol number 2014.08). Written informed consent was obtained from all participants.

### Social Cognition and Neuropsychological Assessment

The **Ekman 60 Faces (Ek-60F) test** quantifies the participant’s ability to recognise six basic human emotions from faces: happiness, sadness, anger, disgust, fear and surprise. The assessment consists of 60 black-and-white trials taken from the Picture and Facial Affect series (Ekman and Friesen, 1976). One point is assigned to every correct answer and the global score is calculated by summing up all points for a maximum total of 60. A cut-off value of 37 has been established in a standardised normative sample (Dodich et al., 2014). This test engages recognition of facial expressions that prompts executive-related decision-making processes. These rely on semantic processing of visual cues and verbal descriptions and, in turn, allow labelling and assimilation of others’ affective mental states (Phillips et al., 2010; Circelli et al., 2013).

Secondly, the **Reading the Mind in the Eyes Test** (RMET) consists of 36 close-up photographs of the eye region (Baron-Cohen et al., 2001). The participant is asked to choose the word that best matches the emotion or thinking process reflected in the eyes’ expression among four alternatives. This test provides a valid measure of mentalising skills, in particular of the affective ToM component of emotional state recognition and processing (Serafin and Surian, 2004; Vellante et al., 2013).

Lastly, the **Story-based Empathy Task** (SET) consists of non-verbal cartoon-vignettes designed to assess the ability to attribute intentions (Intention Attribution, SET-IA) or emotional states (Emotion Attribution, SET-EA) to others. SET-IA and SET-EA performance sub-scores were used as proxies of cognitive and affective ToM, respectively. A third sub-section of this test serves as a control condition. The task is composed by a total of 18 story trials, 6 for each sub-task, and the instructions are to choose the most suitable epilogue for each story. The global score (SET-GS) is calculated by summing up all sub-scores (Cerami et al., 2014; Dodich et al., 2015).

Additionally, each participant completed a comprehensive neuropsychological testing battery for a detailed portrayal of cognitive performance. The Mini-Mental State Examination (MMSE) served as an overall indicator of cognitive performance. Memory functioning was assessed with the Prose Memory test (immediate and 10-min delayed recall) and with the Rey-Osterrieth Complex Figure (10-min delayed recall) for verbal and visuo-spatial long-term memory, respectively. The Category Fluency test (3 categories, 1-min each) was used as test of semantic memory. The Verbal Paired Associates Learning test was also administered as an additional measure of verbal memory assessing the interplay between episodic and semantic processing. Semantic processing was further explored via the administration of the WAIS-Similarities sub-test, and lexical recall was assessed with the Confrontation Naming test. The Digit Span test (forward and backward) was then administered to assess short-term and working memory, respectively. Executive functions were examined through the Stroop test to assess inhibition and attention, the Letter Fluency test to assess cognitive control in lexical access and the Raven’s Coloured Progressive Matrices to assess abstract reasoning. The copy of the Rey-Osterrieth Complex Figure was used as a measure of visuo-constructive abilities. Finally, the Token test and the Digit Cancellation test were used as measures of language comprehension and visual selective attention, respectively. The selected cognitive battery had been validated in the local memory clinic, showing to be particularly sensitive to the impairment reflective of the early stages of AD (Wakefield et al., 2014).

### Magnetic Resonance Imaging Acquisition and Processing

Neuroanatomical T1-weighted Turbo Field Echo images were acquired with a Philips Achieva 1.5 T scanner with the following parameters: Voxel size: 1.1 × 1.1 × 0.6 mm^3^; repetition time 7.4 ms; echo delay time 3.4 ms; flip angle 8°; field of view 250 mm; matrix size 256 × 256 × 124. Functional resting-state Echo-planar images were acquired with the following parameters: Voxel size: 3.28 × 3.28 × 6.00 mm^3^; repetition time 2 s; echo delay time 50 ms; flip angle 90°; field of view 230 mm; matrix size 64 × 64. Two hundred and forty volumes were acquired, preceded by 10 dummy scans to allow the scanner to reach equilibrium; each volume consisted of 20 slices acquired axially and contiguously, in ascending order.

Structural voxel-based morphometry (VBM) analysis was carried out with the most updated standard VBM procedures available in Statistical Parametric Mapping (SPM) 12 software (Wellcome Centre for Human Neuroimaging, London, United Kingdom). Firstly, scans were reoriented and segmented into grey matter, white matter and cerebrospinal fluid tissue density maps. Secondly, images were normalised to the Montreal Neurological Institute (MNI) space and modulated. Lastly, images were smoothed with an 8-mm full-width at half maximum Gaussian kernel. Quantification of global tissue map volumes was carried out with the get_totals script^1^ to calculate total intracranial volumes and thus account for overall head size variability (Peelle et al., 2012). Hippocampal volumes were obtained through the STEPS automated process^2^ that allows accurate multi-template segmentation of bilateral grey-matter hippocampal contours from the native-space T1-weighted images (Jorge Cardoso et al., 2013).

Resting-state functional scans were pre-processed via a standard pipeline (Postema et al., 2019) that included the following steps: slice timing, to standardise single-subject time-related discrepancies within each volume; spatial realignment to adjust for linear and rotational head motion; spatial normalisation of images to an echo-planar imaging template (during which voxel size was resized to 2 mm^3^); temporal filtering (0.01 – 0.1 Hz) to reduce artefact-related signal not associated with neural activity; and, finally, a 6-mm Gaussian kernel smoothing to maximise signal-to-noise ratio. Images were then elaborated with an independent component analysis (ICA) to extract functional connectivity patterns reflecting major large-scale brain networks (Beckmann et al., 2005). The GIFT toolbox (v1.3i^3^) was used for this purpose. The Infomax optimisation algorithm was chosen and the number of components to be extracted was set to 20, as a reliable amount that typically allows the extraction of the fundamental resting-state human connectivity networks (Wang and Li, 2015).

Five networks were selected, namely, the anterior DMN (aDMN), the posterior DMN (pDMN), the left and right fronto-parietal networks and the salience network, due to their established involvement in cognitive functioning (Bressler and Menon, 2010). Component selection was carried out independently (with 100% agreement) by three raters (JMVB, MDM and AV) based on the visual recognition of their topographical features. Sources of variability, such as in-scanner motion parameters, were discarded during the ICA process by separating maps that represent signal dependent neural networks from artefact-related components.

### Statistical Analyses

Demographics, social cognition and basic neuropsychological scores were analysed with IBM SPSS Statistics 24 software for Windows (SPSS Inc., Chicago, IL, United States). The Shapiro-Wilk test for normality performed on the residuals of the outcome variables of interest showed non-normal distributions for most of the models. Firstly, Mann-Whitney U statistics was used to compare the neuropsychological profile of our sample and that of the healthy matched control group. A non-parametric correlation model was run to establish the degree of association among the various social sub-scores and quantify inter-test reliability. Subsequently, a non-parametric partial correlation analysis was carried out between social cognition and basic neuropsychological scores. Significance was set at a Bonferroni-corrected p (0.05/15) = 0.003.

An initial structural VBM whole brain *t*-test analysis was carried out between the scans of the patient sample and those of the sample of healthy matched controls. Voxel-based multiple regression statistical models were then carried out testing the association between social cognition indices and cerebral grey-matter maps. An uncorrected cluster-forming threshold of *p* < 0.005 was used and clusters surviving a Family Wise Error (FWE) corrected threshold of *p* < 0.05 were retained as significant. Peak region coordinates were transformed from MNI space to Talairach stereotactic space with the Talairach client software and Lancaster transformation method (Lancaster et al., 1997, 2000). To maximise consistency, all extracted large-scale network maps were modelled independently as a function of the target proxies of social cognition, along the same methodological lines as in the voxel-based morphometry multiple regressions. Only patients were included in the social cognition correlation models.

All inferential imaging models in the present study were controlled for age (Fox and Schott, 2004); years of education, as a proxy of cognitive reserve (Fratiglioni and Wang, 2007); total intracranial volume, to account for head size (Peelle et al., 2012), and normalised hippocampal volumes (Jorge Cardoso et al., 2013), to control for a distinctive marker of regional disease-related degeneration. Neuropsychological partial correlation models were controlled for age, years of education and normalised hippocampal volumes. Details on exact p-values adopted in each set of inferential models are reported at the bottom of each table.

## Results

**FIGURE 1:**
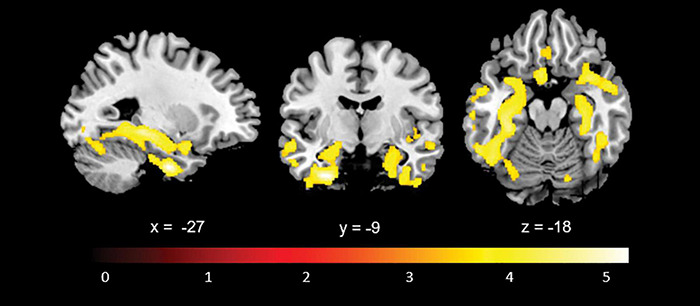
Statistical whole-brain grey matter volumetric comparison between the participant sample (*n* = 46) and a control group (*n* = 34) showing atrophy in medial temporal lobe structures consistent with a probable AD aetiology.

Whole brain comparison of baseline structural scans of all patients with structural scans of age- and education-matched healthy controls (mean age 74.73, SD 6.96, *p* = 0.73; mean years of education 9.94, SD 3.99, *p* = 0.60) showed smaller grey matter volumes in bilateral medial temporal cortices, supportive of a probable AD aetiology (Figure 1).

Similar comparisons of functional brain networks maps showed a profile of functional alterations typical of probable AD (see Supplementary Material, Supplementary Figures 1, 2, and Supplementary Table 1 for a detailed description and graphical representation of these results).

### Associations Between Social Cognition and Neuropsychological Scores

Demographic and neuropsychological profile details are summarised in Table 1. Statistical comparisons showed group differences compatible with a cognitive decline of the probable AD type.

**TABLE 1:** Demographic characteristics and cognitive profile of the patient sample (*n* = 46) and healthy sample.

Variable	Patient sample (*n* = 46) Mean/(SD)	Healthy sample (*n* = 34) Mean/(SD)	p-value
**Demographic data**			
Gender, *n* (%)	17 M (37%): 29 F (63%)	16 M (47%): 18 F (53%)	0.52
Age (years)	75.33 (7)	74.73 (6.96)	0.73
Years of education	9.54 (3.86)	9.94 (3.99)	0.60
**Neuropsychological data**			
MMSE	25.95 (2.61)	28.73 (1.39)	0.001*
Letter fluency	26.65 (12.24)	32.70 (11.23)	0.023*
Category fluency	27.61 (12.33)	36.64 (8.89)	0.001*
Prose memory immediate	6.61 (3.69)	9.91 (3.30)	0.001*
Prose memory delayed	7.09 (5.20)	12.79 (4.68)	0.001*
Rey-Osterrieth Complex Figure copy	25.27 (9.07)	32.23 (3.19)	0.001*
Rey-Osterrieth Complex Figure recall	6.15 (4.87)	13.42 (5.33)	0.001*
Digit span forward	5.33 (0.84)	5.70 (0.90)	0.05*
Digit span backward	3.66 (0.99)	3.85 (0.82)	0.20
Digit cancellation	42.65 (9.99)	50.26 (7.14)	0.001*
WAIS-Similarities	14.85 (4.66)	19.82 (4.47)	0.001*
Verbal Paired Associates Learning	8.05 (3.85)	11.63 (3.44)	0.001*
Confrontation naming	16.58 (3.33)	18.38 (2.01)	0.004*
Token test	31.43 (2.70)	34.12 (1.98)	0.001*
Raven’s Coloured Progressive Matrices	23.67 (5.97)	28.44 (4.0)	0.001*

*Non-parametric Mann-Whitney U significant results reported as p < 0.05. MMSE: Mini Mental State Examination. M: Male. F: Female.

All social cognition scores were correlated among each other (Table 2). Associations between social cognition scores and basic neuropsychological scores are displayed in Table 3. Overall cognitive performance, measured with the MMSE, was positively correlated with the RMET (ρ = 0.518, *p* = 0.001) and the Ek-60F tests (ρ = 0.554, *p* = 0.001). The RMET also correlated with performance on the Letter Fluency test (ρ = 0.607, *p* = 0.001), Category Fluency test (ρ = 0.631, *p* = 0.001), Digit Cancellation test (ρ = 0.577, *p* = 0.001), Raven’s Coloured Progressive Matrices (ρ = 0.450, *p* = 0.002), WAIS Similarities test (ρ = 0.491, *p* = 0.001), the Digit Span Backward (ρ = 0.447, *p* = 0.003) and the Confrontation Naming test (ρ = 0.486, *p* = 0.001). The Ek-60F test was positively correlated with the Letter Fluency test (ρ = 0.442, *p* = 0.003), Digit Cancellation test (ρ = 0.551, *p* = 0.001), Digit Span Backward (ρ = 0.439, *p* = 0.003) and with the Token test (ρ = 0.496, *p* = 0.001). Finally, none of the SET scores showed significant correlations with scores on neuropsychological tests.

**TABLE 2:** Non-parametric correlations among social cognition measures.

Social cognition inter-domain correlations				

	**Mean/(SD)**	**RMET**	**Ek-60F**	**SET-GS**
RMET	19.89/(5.86)	-		
Ek-60F	39.80/(8.95)	ρ = 0.672/*p* = 0.001*	-	
SET – GS	13.48/(3.55)	ρ = 0.348/*p* = 0.012*	ρ = 0.493/*p* = 0.001*	-

*Significant results are reported at a p (0.05/3) < 0.017 after correction for multiple comparisons.

*Ek-60F: Ekman 60 Faces test; RMET: Reading the Mind in the Eyes Test; SD: Standard deviation; SET-GS: Story-based Empathy Task Global Score. Correlation models were controlled for age, years of education and normalised hippocampal volume ratio.*

**TABLE 3:** Non-parametric correlations between social cognition and neuropsychological measures.

Social cognition correlations			

	**RMET^a^**	**Ek-60F^a^**	**SET-GS^ab^**
MMSE	ρ = 0.518/*p* = 0.001*	ρ = 0.554/*p* = 0.001*	ρ = 0.404/*p* = 0.007
Letter fluency	ρ = 0.607/*p* = 0.001*	ρ = 0.442/*p* = 0.003*	ρ = 0.361/*p* = 0.017
Category fluency	ρ = 0.631/*p* = 0.001*	ρ = 0.301/*p* = 0.050	ρ = 0.333/*p* = 0.029
Prose memory immediate	ρ = 0.193/*p* = 0.215	ρ = 0.043/*p* = 0.786	ρ = 0.251/*p* = 0.105
Prose memory delayed	ρ = 0.345/*p* = 0.023	ρ = 0.246/*p* = 0.112	ρ = 0.167/*p* = 0.283
Rey-Osterrieth Complex Figure copy	ρ = 0.400/*p* = 0.008	ρ = 0.197/*p* = 0.205	ρ = 0.218/*p* = 0.160
Rey-Osterrieth Complex Figure recall	ρ = 0.187/*p* = 0.230	ρ = 0.057/*p* = 0.715	ρ = 0.011/*p* = 0.946
Digit span forward	ρ = 0.299/*p* = 0.052	ρ = 0.399/*p* = 0.008	ρ = 0.361/*p* = 0.017
Digit span backward	ρ = 0.447/*p* = 0.003*	ρ = 0.439/*p* = 0.003*	ρ = 0.425/*p* = 0.004
Digit cancellation	ρ = 0.577/*p* = 0.001*	ρ = 0.551/*p* = 0.001*	ρ = 0.388/*p* = 0.010
WAIS-Similarities	ρ = 0.491/*p* = 0.001*	ρ = 0.330/*p* = 0.031	ρ = 0.245/*p* = 0.113
Verbal Paired Associates Learning	ρ = 0.199/*p* = 0.200	ρ = 0.077/p = 0.624	ρ = 0.054/*p* = 0.732
Confrontation naming	ρ = 0.486/*p* = 0.001*	ρ = 0.338/*p* = 0.027	ρ = 0.247/*p* = 0.110
Token test	ρ = 0.383/*p* = 0.011	ρ = 0.496/*p* = 0.001*	ρ = 0.209/*p* = 0.178
Raven’s Coloured Progressive Matrices	ρ = 0.450/*p* = 0.002*	ρ = 0.273/*p* = 0.076	ρ = 0.299/*p* = 0.051

*Significant results are only reported as p (0.05/15) < 0.003 after correction for multiple comparisons.

*^a^Correlation models were controlled for age, years of education and normalised hippocampal volume.*

*^b^The SET-GS test showed no significant correlations.*

*Ek-60F: Ekman 60 Faces test; MMSE: Mini-Mental State Examination; RMET: Reading the Mind in the Eyes Test; SD: Standard deviation; SET-GS: Story-based empathy task global.*

### Association Between Social Cognition Scores and Grey Matter Volume

**FIGURE 2:**
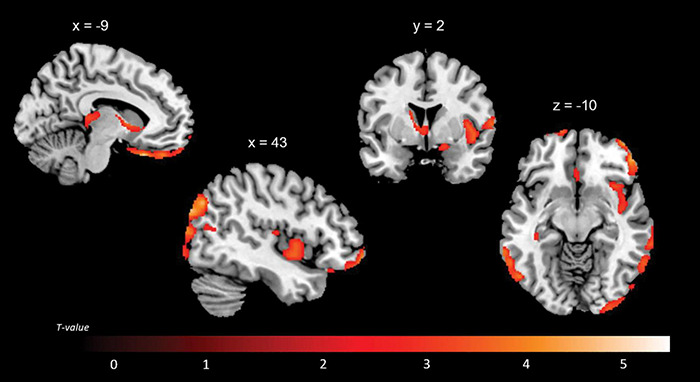
Positive correlations between residual grey matter volume and RMET outcomes.

**FIGURE 3:**
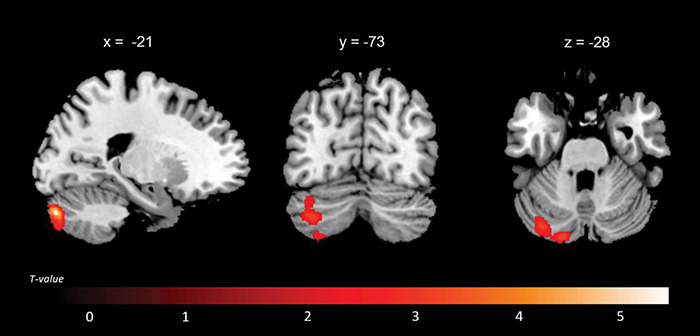
Regions that displayed a positive correlation between the SET-EA scores and grey matter volume.

A positive association between RMET scores and grey matter volume was found in the left anterior cingulate cortex (ACC), orbitofrontal cortex (OFC) (BA11), middle temporal gyrus, middle occipital gyrus, thalamus, caudate and cerebellum; in the right inferior lateral frontal cortex, inferior and middle temporal gyri, temporoparietal junction (BA39), superior occipital gyrus; and in the bilateral superior temporal sulcus (BA21/22) (Figure 2 and Table 4). While no significant results emerged from the analysis of SET-GS scores, performance on the SET-EA sub-test was positively associated with grey matter in the left cerebellum (Figure 3 and Table 5). No significant volumetric associations were found with the Ek-60F test.

**TABLE 4:** Grey matter clusters of significant correlation for the RMET.

Peak-based localisation (BA)^a^	HS	Cluster extent	FWE corrected *p*-value^*^	*Z* Score	MNI coordinates		
					X	y	z
Anterior cingulate cortex (BA 32)	L	6854	0.001	4.56	–20	21	–30
Inferior frontal gyrus (BA 45)	R			4.52	58	33	4
Rectal gyrus (BA 11)	L			4.20	–10	22	–28
Middle temporal gyrus (BA 21)	R	1750	0.009	4.46	69	–28	–21
Inferior temporal gyrus (BA 20)	R			3.86	62	–44	–27
Inferior temporal gyrus (BA 21)	R			3.35	63	–54	–8
Superior occipital gyrus (BA 19)	R	3918	0.001	4.46	42	–82	27
Angular gyrus (BA 39)	R			4.30	48	–72	32
Middle temporal gyrus (BA 19)	R			4.14	52	–80	6
Middle temporal gyrus (BA 22)	L	2473	0.002	3.99	–69	–44	0
Cerebellum	L			3.79	–51	–42	–33
Middle occipital gyrus (BA 37)	L			3.60	–54	–66	–12
Thalamus	L	1554	0.013	3.76	–14	–33	4
Caudate	L			3.43	–12	9	10

*Threshold of significance defined at p = 0.005.

*^a^Inferior frontal lateral cortex: Brodmann area 45. Orbitofrontal cortex: Brodmann area 11. Superior temporal sulcus: Brodmann areas 21/22. Temporoparietal junction: Brodmann area 39.*

*BA: Brodmann area; FWE: Family Wise Error; HS: Hemispheric side; L: Left; MNI: Montreal Neurological Institute; R: Right; RMET: Reading the Mind in the Eye Test.*

**TABLE 5:** Grey matter clusters of significant correlation for the SET-EA.

Peak-based localisation	HS	Cluster extent	FWE corrected *p*-value^*^	*Z* Score	MNI coordinates		
					x	y	z
Cerebellum: Uvula	L	2167	0.004	5.03	–20	–86	–33
Cerebellum: Declive	L			3.40	–33	–78	–28
Cerebellum	L			2.81	–28	–75	–52

*Threshold of significance defined at p = 0.005.

*FWE: Family Wise Error; HS: Hemispheric side; L: Left; MNI: Montreal Neurological Institute; SET-EA: Story-based Empathy task: Emotion Attribution sub-task.*

### Associations Between Social Cognition Scores and Resting-State Brain MRI Function

**FIGURE 4:**
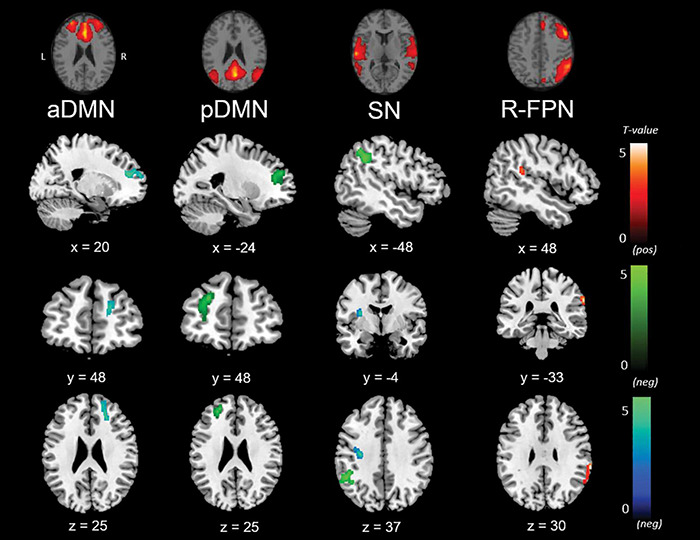
Negative (Ek-60F, green; RMET, blue), and positive (Ek-60F, red) correlations between functional connectivity of the anterior default mode network (aDMN), posterior default mode network (pDMN), salience network (SN), right fronto-parietal network (r-FPN) and social cognition scores.

Outcomes from the multiple regression models between large-scale network connectivity maps and social cognition scores are displayed in Figure 4 and Table 6. No significant results were found with the SET-GS or any of its sub-tests.

**TABLE 6:** Clusters of significant correlation between social cognition scores and functional connectivity of the aDMN, r-FPN and salience network.

Peak-based localisation (BA)	HS	Cluster extent	FWE corrected *p*-value^*^	*Z* Score	MNI coordinates		
					x	y	z
**Right fronto-parietal network (r-FPN)**							

**Ek-60F (+)**							
Inferior parietal lobule (BA 40)	R	243	0.049	4.65	66	–36	32
Angular gyrus (BA 39)	R			3.37	54	–62	26
Insula (BA 13)	R			3.56	50	–38	22

**Anterior default mode network (aDMN)**							

**RMET (-)**							
Middle frontal gyrus (BA 9)	R	256	0.040	4.06	24	34	24
Superior frontal gyrus (BA 10)	R			3.75	22	58	20
Middle frontal gyrus (BA 9)	R			3.72	22	42	20

**Posterior default mode network (pDMN)**							

**Ek-60F (-)**							
Precentral gyrus (BA 4)	L	507	0.001	5.07	–48	–12	42
Precentral gyrus (BA 6)	L			3.74	–50	–6	34
Precentral gyrus (BA 6)	L			3.34	–34	–16	38
Superior frontal gyrus (BA 10)	L	411	0.002	4.86	–20	52	28
Middle frontal gyrus (BA 10)	L			4.63	–28	44	18

**Salience network**							

**Ek-60F (-)**							
Supramarginal gyrus (BA 40)	L	275	0.024	3.83	–50	–48	36
Supramarginal gyrus (BA 40)	L			3.56	–44	–42	34
Inferior parietal lobule (BA 40)	L			3.56	–48	–52	44
**RMET (-)**							
Precentral gyrus (BA 6)	L	342	0.007	5.23	–32	–16	34
Insula (BA 13)	L			3.47	–38	–8	26
Claustrum	L			4.50	–30	–6	16

*Threshold of significance defined at p = 0.005.

*(-) = negative correlation; (+) = positive correlation; BA: Brodmann area; Ek-60F: Ekman 60 Faces test; FWE: Family Wise Error; HS: Hemispheric side; L: Left; MNI: Montreal Neurological Institute; R: Right; RMET: Reading the Mind in the Eye Test.*

Firstly, the Ek-60F test showed a significant positive association with strength of functional connectivity of the right fronto-parietal network (r-FPN) in the right insula (BA13) and TPJ (BA39/40). In contrast, this test displayed a negative association with connectivity of the salience network in the left TPJ (BA40) and of the pDMN in the left precentral gyrus (BA6) and anterior prefrontal cortex (BA10). Similarly, a negative association was found between the RMET and functional connectivity of the aDMN in the right anterior prefrontal cortex (BA9/10) and of the salience network in the left precentral gyrus (BA6), claustrum and insula (BA13).

## Discussion

This study established the neuropsychological, structural and functional connectivity associations of social cognition and ToM abilities in the prodromal to mild stages of probable AD.

### Associations Between Social Cognition Skills and Neuropsychological Profiling

Significant positive associations were found between neuropsychological profiles and the Ek-60F and RMET, proxies measures of recognition and processing of affective mental states (Valle et al., 2015). Performance on both tests was associated with overall cognitive levels, working-memory, executive functions and selective attention scores. A link between socio-cognitive abilities and global cognition has been consistently documented in AD (Cuerva et al., 2001; Phillips et al., 2010; Freedman et al., 2013; Torres et al., 2015). Selective attention is central to social cognition, as it supports decision-making, elicited by processing of verbal information that is typical of social interactions and by visual recognition and labelling of facial expressions that are central to activating affective connotations (Phillips et al., 2010; García-Rodríguez et al., 2012; Circelli et al., 2013; Hot et al., 2013). Therefore, selective attention modulates responses arising from mental representations, including those of others (Leslie et al., 2004). Moreover, memory decline in AD may dictate a need for increased supply of attentional and executive resources that would have to be channelled toward independent cognitive processes occurring concomitantly during ToM tasks (García-Rodríguez et al., 2012).

Performance on the Ek-60F was also correlated with verbal comprehension, while the RMET showed associations with proxies of semantic memory, language and executive functions. Semantic memory mechanisms are essential for recognising and attributing mental states in unfamiliar environments (Ciaramelli et al., 2013). The association between social cognition and aspects of language, semantic memory, comprehension and reasoning may reflect the early ontogenesis of mentalising abilities in support of communication during childhood development (Miller, 2006). The inherent and indissoluble link between communication and the social need of human collaboration is of aid to understand the patients’ functional decline (Falk and Bassett, 2017). A deteriorating relation between non-verbal affective processing and verbal communication, in fact, may signpost the onset of social disconnection between patient and carer, and may lead to build-up of burden in the carer (Martinez et al., 2018).

Our data provide support to the premise that executive functions are closely linked to social cognition in AD (Ramanan et al., 2017; Lucena et al., 2020). Firstly, performance on the WAIS-Similarities test, which relies on verbal reasoning but is also supported by executive processes (Woo et al., 2010), was shown to be associated with affective ToM (Miguel et al., 2017). Moreover, verbal reasoning is linked to cognitive ToM scores when AD patients are asked to solve tasks based on false beliefs (Zaitchik et al., 2004; Takenoshita et al., 2018). Secondly, verbal fluency is associated with affective ToM in non-clinical populations (Saltzman et al., 2000; Ahmed and Stephen Miller, 2011), and AD (Laisney et al., 2013; Chainay and Gaubert, 2020; Yildirim et al., 2020). Lastly, abstract reasoning is also considered a proxy of executive functions (Diamond, 2013), and has been associated with ToM in AD (Cuerva et al., 2001). Consolidation of reasoning and attentional functions may contribute to shape the internal thoughts used to create social inferential representations linked to self-awareness (Demetriou et al., 2018).

### Associations Between Social Cognition Skills and Brain Structure

The significant findings that emerged from the model of affective ToM scores (RMET) showed consistency with known neuroanatomical ToM core regions (Abu-Akel and Shamay-Tsoory, 2011; Schurz et al., 2014; Van Overwalle et al., 2014).

Emerged as a crucial region associated with performance on the RMET, the left ACC shows higher functional task-based activation during ToM performance in MCI individuals compared to controls (Baglio et al., 2012). Similarly, Sapey-Triomphe et al. (2015) showed positive associations between volumes of the left ACC and right OFC and proxies of affective processing measured through an emotion recognition task. In early-AD, alterations in the left ACC underpin deterioration in self-awareness (Amanzio et al., 2011; Valera-Bermejo et al., 2020), and sustain the creation of complex self-other brain representations (Amodio and Frith, 2006). In addition to the ACC, affective ToM was also associated with the left OFC. Since patients presenting with selective focal damage of this region manifest affective ToM deficits, it has been suggested that this structure might be involved in social-related decision making (Jonker et al., 2017). Lastly, the inferior frontal cortex, detected in our results, has been found to show activation during affective ToM tasks in healthy individuals (Schlaffke et al., 2015).

The right TPJ displayed the largest association with affective ToM in the present study, an area considered essential for ToM abilities (Saxe and Wexler, 2005; Perner et al., 2006; Aichhorn et al., 2009; Schurz et al., 2014; Krall et al., 2015). Integrity of the right TPJ, which in its parietal portion is also a key node of the DMN, has shown to be predictive of ToM thinking in ageing (Hughes et al., 2019). The contralateral portion of the TPJ has also been particularly associated with ToM in AD. Dermody et al. (2016) reported an association between grey matter in the left TPJ and assessment of perspective-taking empathetic processing in AD patients. Likewise, Kumfor et al. (2017) found similar associations when evaluating emotions, including clusters within the left TPJ. Moreover, hypometabolism in the left TPJ is greater in AD patients than in fronto-temporal dementia in relation to ToM performance (Le Bouc et al., 2012).

Prefrontal and parietal areas may contribute, conjointly, to the processes of self-perspective inhibition (for which the frontal cortex may play a major role), and of affective recognition by integrating inferential representations and creating attribution of others’ beliefs (that would be instead sustained by the TPJ) (Saxe and Kanwisher, 2003; Le Bouc et al., 2012). In this context, patients with prefrontal or temporoparietal lesions have been shown to underperform during social cognition and ToM tasks (Rowe et al., 2001; Samson et al., 2004).

Associations with the occipital cortex have been evidenced during ToM performance (Otti et al., 2015), and they may reflect a prerequisite visual attribute processing to initiate affective processing. Lastly, subcortical bilateral volumetric associations between the cerebellum and affective ToM (SET-EA) provide insights on the cumulative research that demonstrates the substantial contribution of cerebellar cortices to social cognition abilities (Schmahmann, 2019), a contribution that seems to be crucial for high level abstraction, mirrored-based motor tasks and executive processing (Van Overwalle et al., 2014). In AD patients, there has been significant evidence of cerebellar implications in ToM functions, possibly as a structure that supports cognitive coordination during switching between one’s own and others’ mental states (Baglio et al., 2012; Dermody et al., 2016; Synn et al., 2018).

### Associations Between Social Cognition Skills and Brain Function

Patterns of resting-state connectivity showed associations with affective recognition and processing in the main large-scale networks supportive of cognition: DMN, r-FPN (central executive) and salience network (Bressler and Menon, 2010). In early-AD, selective pathological vulnerability is shown by the DMN (Greicius et al., 2004; Broyd et al., 2009; Eyler et al., 2019), that is the network most tightly associated with ToM performance (Mars et al., 2012; Li et al., 2014).

Firstly, scores of emotion recognition and processing (Ek-60F and RMET) were negatively correlated with strength of functional connectivity of the anterior and posterior DMN in the left and right dorsomedial prefrontal cortex, respectively. In this context, social cognition impairment has been found in patients with mPFC lesions (Bird et al., 2004). In early AD, decreased DMN connectivity within the mPFC might reflect redistribution of cognitive resources to other cognitive networks that support social cognitive functions.

Secondly, functional connectivity within the salience network showed a negative association with emotion recognition and processing (Ek-60F and RMET) in the left TPJ, left precentral gyrus and insula. In this context, previous research has shown that the left TPJ displays less connectivity during processing of salient stimuli (Kucyi et al., 2012). Additionally, impairment in the left TPJ has been associated with reduced mentalising performance in AD (Le Bouc et al., 2012; Dermody et al., 2016; Kumfor et al., 2017). Therefore, the salience network, essential in brain functional organisation of internally/externally directed thought processes (Corbetta et al., 2008) might have a modulatory role by down-regulating inter-network coupling with the left TPJ to foster functional shifting and facilitate increasing of connectivity of other networks harboured within the right TPJ. Networks in charge of modulating self-internal/others-external attentional resources could adapt to sustain social cognition performance (Lieberman, 2007).

Lastly, connectivity strength within the r-FPN showed a positive association with emotion recognition (Ek-60F) in the right TPJ and right insula. Increased bilateral activation of these regions has been linked to affective and facial recognition (Fusar-Poli et al., 2009), in addition to mentalising (Saxe and Wexler, 2005). Our results shed light on the involvement of the TPJ and insular cortex into a network supporting affective recognition and processing during social cognition. This intercommunication between social cognitive and executive networks accentuates the crucial contribution of executive functional resources in support of affective processing. In the context of AD, Chen et al. (2019) found stronger functional coupling between the salience and fronto-parietal network in MCI patients. In addition, hypermodulation of the central executive fronto-parietal network in the context of salience alterations has been found in the MCI population (Chand et al., 2017). Our results showed that lower connectivity of the left insula in the salience network, but higher inter-network connectivity of the right insula with the right fronto-parietal network, were associated with social cognition performance. Stronger inter-network connectivity between the insular hub of the salience network and the executive fronto-parietal network may be explained in the context of a dysfunctional DMN in early-AD. The fronto-parietal network has been proposed to serve as a supplementary system ancillary to the DMN in the regulation of introspection and self-awareness based on executive reasoning of complex social representations (Dixon et al., 2018). These modulations in network dynamics might reflect a combination of adaptive and maladaptive processes at play in support of behavioural response in a system grossly depleted by advancing neurodegeneration.

### Limitations

A possible limitation may arise from the choice of combining patients with different disease severity levels. To account for differences in disease severity, a correction factor was included in the analyses to control for the influence of severity of neurodegeneration, i.e., a proxy of hippocampal integrity. A second potential limitation may be our decision to implement three different social cognitive measures that might not rely on shared neural substrates. The presence of variable results across the three instruments, however, may reflect the heterogeneous nature of affective abilities and ToM, whereby the outcome of the assessments is complementary rather than capturing a single construct.

## Conclusion

In summary, our results support a modular “social cognitive network” that relies on multiple-network intercommunication while engaging in social-cognitive tasks (Chiong et al., 2013). Based on the present cognitive and neuroimaging data, we suggest that patients in the prodromal to mild stages of probable AD rely significantly on executive resources to sustain affective recognition and processing as a possible adaptive effect to support behavioural performance in response to neurodegeneration. This finding could provide insights about the lack of substantial social deficits in early-AD. Brain executive networks, which are expressed in neural territories relatively spared by AD pathology in the early disease phases, may compensate for network dysfunction affecting those systems sustaining mentalisation, i.e., inherent DMN breakdown, providing the necessary attentional/executive support to sustain the attribution of self-other representations.

Differences in brain laterality, evidenced in regions associated with affective social processing (Schurz et al., 2014), demonstrated lower functional connectivity in the left insula and TPJ but stronger connectivity in the right insula and TPJ, establishing, therefore, the essential contribution of right-sided brain resources for optimal socio-affective performance in early-AD patients. We propose that the right insular cortex, an integrative core region of emotion recognition and processing (Kurth et al., 2010), may function as a structure responsible for affective modulation that arbitrates network coupling in the context of a possible compensatory up-regulation of the central executive network during ToM performance, supported by increased connectivity in the right TPJ. The characterisation of a social cognitive profile in early-AD could provide insights on the impact of neurodegeneration over social cognition networks and provide a supportive explanation for the heterogeneity of behavioural, structural and functional social cognition results in AD compared with other neurodegenerative conditions (Poletti et al., 2012; Christidi et al., 2018; Cotter et al., 2018).

## Data Availability Statement

The raw data supporting the conclusions of this article will be made available by the authors, without undue reservation.

## Ethics Statement

The studies involving human participants were reviewed and approved by the Health and Care Research Wales (HCRW) Committee. The patients/participants provided their written informed consent to participate in this study.

## Author Contributions

JMVB contributed to data extraction and curation, data analyses, result interpretation, and writing of the initial draft of the manuscript. MDM contributed to data collection, result interpretation, and critical revision of the manuscript. MM contributed to data collection and critical revision of manuscript. CC contributed to study conception, result interpretation, and critical revision of manuscript. AD contributed to critical revision of manuscript. AV contributed to study conception, clinical assessment and diagnosis, and revising and finalising of the manuscript. All authors contributed to the article and approved the submitted version.

## Conflict of Interest

The authors declare that the research was conducted in the absence of any commercial or financial relationships that could be construed as a potential conflict of interest.

## Publisher’s Note

All claims expressed in this article are solely those of the authors and do not necessarily represent those of their affiliated organizations, or those of the publisher, the editors and the reviewers. Any product that may be evaluated in this article, or claim that may be made by its manufacturer, is not guaranteed or endorsed by the publisher.

## Footnotes

1
http://www0.cs.ucl.ac.uk/staff/g.ridgway/vbm/get_totals.m

2
http://cmictig.cs.ucl.ac.uk/nifyweb/

3
https://trendscenter.org/software/gift/

## References

[B1] Abu-Akel A. Shamay-Tsoory S. (2011). Neuroanatomical and neurochemical bases of theory of mind. *Neuropsychologia* 49 2971–2984. 10.1016/j.neuropsychologia.2011.07.012 21803062

[B2] Ahmed F. S. Stephen Miller L. (2011). Executive function mechanisms of theory of mind. *J. Autism Dev. Dis.* 41 667–678. 10.1007/s10803-010-1087-7 20811770

[B3] Aichhorn M. Perner J. Weiss B. Kronbichler M. Staffen W. Ladurner G. (2009). Temporo-parietal junction activity in theory-of-mind tasks: falseness, beliefs, or attention. *J. Cogn. Neurosci.* 21 1179–1192. 10.1162/jocn.2009.21082 18702587

[B4] Albert M. S. DeKosky S. T. Dickson D. Dubois B. Feldman H. H. Fox N. C. (2011). The diagnosis of mild cognitive impairment due to Alzheimer’s disease: recommendations from the National Institute on Aging-Alzheimer’s Association workgroups on diagnostic guidelines for Alzheimer’s disease. *Alzheimers Demen.* 7 270–279. 10.1016/j.jalz.2011.03.008 21514249 PMC3312027

[B5] Amanzio M. Torta D. M. E. Sacco K. Cauda F. D’Agata F. Duca S. (2011). Unawareness of deficits in Alzheimer’s disease: role of the cingulate cortex. *Brain* 134 1061–1076. 10.1093/brain/awr020 21385751

[B6] Amodio D. M. Frith C. D. (2006). Meeting of minds: the medial frontal cortex and social cognition. *Nat. Rev. Neurosci.* 7:268. 10.1038/nrn1884 16552413

[B7] Badhwar A. Tam A. Dansereau C. Orban P. Hoffstaedter F. Bellec P. (2017). Resting-state network dysfunction in Alzheimer’s disease: A systematic review and meta-analysis. *Alzheimer’s Demen.* 8 73–85. 10.1016/j.dadm.2017.03.007 28560308 PMC5436069

[B8] Baglio F. Castelli I. Alberoni M. Blasi V. Griffanti L. Falini A. (2012). Theory of mind in amnestic mild cognitive impairment: an FMRI study. *J. Alzheimers Dis.* 29 25–37. 10.3233/jad-2011-111256 22156049

[B9] Baron-Cohen S. Leslie A. M. Frith U. (1985). Does the autistic child have a “theory of mind”? *Cognition* 21 37–46. 2934210 10.1016/0010-0277(85)90022-8

[B10] Baron-Cohen S. Wheelwright S. Hill J. Raste Y. Plumb I. (2001). The “reading the mind in the eyes”. test revised version: a study with normal adults, and adults with asperger syndrome or high-functioning autism. *J. Child Psychol. Psychiatry* 42 241–251. 10.1111/1469-7610.00715 11280420

[B11] Beckmann C. F. DeLuca M. Devlin J. T. Smith S. M. (2005). Investigations into resting-state connectivity using independent component analysis. Philosophical transactions of the Royal Society of London. *Ser. B Biol. Sci.* 360 1001–1013. 10.1098/rstb.2005.1634 16087444 PMC1854918

[B12] Bertoux M. de Souza L. C. O’Callaghan C. Greve A. Sarazin M. Dubois B. (2016). Social cognition deficits: the key to discriminate behavioral variant frontotemporal dementia from Alzheimer’s disease regardless of amnesia? *J. Alzheimers Dis.* 49 1065–1074. 10.3233/jad-150686 26756325

[B13] Bird C. M. Castelli F. Malik O. Frith U. Husain M. (2004). The impact of extensive medial frontal lobe damage on ‘Theory of Mind’ and cognition. *Brain* 127 914–928. 10.1093/brain/awh108 14998913

[B14] Bora E. Yener G. G. (2017). Meta-Analysis of social cognition in mild cognitive impairment. *J. Geriat. Psychiatry Neurol.* 30 206–213. 10.1177/0891988717710337 28639876

[B15] Bora E. Walterfang M. Velakoulis D. (2015). Theory of mind in behavioural-variant frontotemporal dementia and Alzheimer’s disease: a meta-analysis. *J. Neurol. Neurosur. Psychiatry* 86 714–719. 10.1136/jnnp-2014-309445 25595152

[B16] Bressler S. L. Menon V. (2010). Large-scale brain networks in cognition: emerging methods and principles. *Trends Cogn. Sci.* 14 277–290. 10.1016/j.tics.2010.04.004 20493761

[B17] Broyd S. J. Demanuele C. Debener S. Helps S. K. James C. J. Sonuga-Barke E. J. (2009). Default-mode brain dysfunction in mental disorders: a systematic review. *Neurosci. Biobehav. Rev.* 33 279–296. 10.1016/j.neubiorev.2008.09.002 18824195

[B18] Castelli I. Pini A. Alberoni M. Liverta-Sempio O. Baglio F. Massaro D. (2011). Mapping levels of theory of mind in Alzheimer’s disease: A preliminary study. *Aging Ment. Health* 15 157–168. 10.1080/13607863.2010.513038 21140304

[B19] Cerami C. Dodich A. Canessa N. Crespi C. Marcone A. Cortese F. (2014). Neural correlates of empathic impairment in the behavioral variant of frontotemporal dementia. *Alzheimers Demen.* 10 827–834. 10.1016/j.jalz.2014.01.005 24589435

[B20] Chainay H. Gaubert F. (2020). Affective and cognitive theory of mind in Alzheimer’s disease: The role of executive functions. *J. Clin. Experimen. Neuropsychol.* 42 371–386. 10.1080/13803395.2020.1726293 32063090

[B21] Chand G. B. Wu J. Hajjar I. Qiu D. (2017). Interactions of the salience network and its subsystems with the default-mode and the central-executive networks in normal aging and mild cognitive impairment. *Brain Connect.* 7 401–412. 10.1089/brain.2017.0509 28707959 PMC5647507

[B22] Chen H. Li Y. Liu Q. Shi Q. Wang J. Shen H. (2019). Abnormal interactions of the salience network, central executive network, and default-mode network in patients with different cognitive impairment loads caused by leukoaraiosis. *Front. Neural. Circ.* 13:42. 10.3389/fncir.2019.00042 31275116 PMC6592158

[B23] Chiong W. Wilson S. M. D’Esposito M. Kayser A. S. Grossman S. N. Poorzand P. (2013). The salience network causally influences default mode network activity during moral reasoning. *Brain* 136 1929–1941. 10.1093/brain/awt066 23576128 PMC3673466

[B24] Christidi F. Migliaccio R. Santamaría-García H. Santangelo G. Trojsi F. (2018). Social cognition dysfunctions in neurodegenerative diseases: neuroanatomical correlates and clinical implications. *Behav. Neurol.* 2018:1849794. 10.1155/2018/1849794 29854017 PMC5944290

[B25] Ciaramelli E. Bernardi F. Moscovitch M. (2013). Individualized Theory of Mind (iToM): when memory modulates empathy. *Front. Psychol.* 4:4–4. 10.3389/fpsyg.2013.00004 23378839 PMC3561727

[B26] Circelli K. S. Clark U. S. Cronin-Golomb A. (2013). Visual scanning patterns and executive function in relation to facial emotion recognition in aging. *Aging Neuropsychol. Cogn.* 20 148–173. 10.1080/13825585.2012.675427 22616800 PMC3448814

[B27] Corbetta M. Patel G. Shulman G. L. (2008). The reorienting system of the human brain: from environment to theory of mind. *Neuron* 58 306–324. 10.1016/j.neuron.2008.04.017 18466742 PMC2441869

[B28] Cotter J. Granger K. Backx R. Hobbs M. Looi C. Y. Barnett J. H. (2018). Social cognitive dysfunction as a clinical marker: A systematic review of meta-analyses across 30 clinical conditions. *Neurosci. Biobehav. Rev.* 84 92–99. 10.1016/j.neubiorev.2017.11.014 29175518

[B29] Cuerva A. G. Sabe L. Kuzis G. Tiberti C. Dorrego F. Starkstein S. E. (2001). Theory of mind and pragmatic abilities in dementia. *Neuropsy. Neuropsychol. Behav. Neurol.* 14 153–158. 11513098

[B30] Demetriou A. Makris N. Kazi S. Spanoudis G. Shayer M. (2018). The developmental trinity of mind: Cognizance, executive control, and reasoning. *Wiley Interdiscip. Rev. Cogn. Sci.* 9:e1461. 10.1002/wcs.1461 29350832

[B31] Dermody N. Wong S. Ahmed R. Piguet O. Hodges J. R. Irish M. (2016). Uncovering the neural bases of cognitive and affective empathy deficits in Alzheimer’s disease and the behavioral-variant of frontotemporal dementia. *J. Alzheimers Dis.* 53 801–816. 10.3233/jad-160175 27258418

[B32] Diamond A. (2013). Executive functions. *Ann. Rev. Psychol.* 64 135–168. 10.1146/annurev-psych-113011-143750 23020641 PMC4084861

[B33] Dixon M. L. De La Vega A. Mills C. Andrews-Hanna J. Spreng R. N. Cole M. W. (2018). Heterogeneity within the frontoparietal control network and its relationship to the default and dorsal attention networks. *Proc. Nat. Acad. Sci. USA* 115 1598–1607. 10.1073/pnas.1715766115 29382744 PMC5816169

[B34] Dodich A. Cerami C. Canessa N. Crespi C. Iannaccone S. Marcone A. (2015). A novel task assessing intention and emotion attribution: Italian standardization and normative data of the Story-based Empathy Task. *Neurol. Sci.* 36 1907–1912. 10.1007/s10072-015-2281-3 26072203

[B35] Dodich A. Cerami C. Canessa N. Crespi C. Marcone A. Arpone M. (2014). Emotion recognition from facial expressions: a normative study of the Ekman 60-Faces Test in the Italian population. *Neurol. Sci.* 35 1015–1021. 10.1007/s10072-014-1631-x 24442557

[B36] Dodich A. Cerami C. Cappa S. F. Marcone A. Golzi V. Zamboni M. (2018). Combined socio-behavioral evaluation improves the differential diagnosis between the behavioral variant of frontotemporal dementia and Alzheimer’s disease: in search of neuropsychological markers. *J. Alzheimers Dis.* 61 761–772. 10.3233/jad-170650 29254091

[B37] Dodich A. Cerami C. Crespi C. Canessa N. Lettieri G. Iannaccone S. (2016). Differential impairment of cognitive and affective mentalizing abilities in neurodegenerative dementias: evidence from behavioral variant of frontotemporal dementia. Alzheimer’s disease, and mild cognitive impairment. *J. Alzheimers Dis.* 50 1011–1022. 10.3233/jad-150605 26836153

[B38] Dvash J. Shamay-Tsoory S. G. (2014). Theory of Mind and empathy as multidimensional constructs: neurological foundations. *Topic Lang. Disord.* 34:40. 10.1097/TLD.0000000000000040

[B39] Ekman P. Friesen W. V. (1976). *Pictures of facial affect.* PaloAlto, CA: Consulting Psychologists Press.

[B40] Eyler L. T. Elman J. A. Hatton S. N. Gough S. Mischel A. K. Hagler D. J. (2019). Resting state abnormalities of the default mode network in mild cognitive impairment: a systematic review and meta-analysis. *J. Alzheimer’s Dis.* 70 107–120. 10.3233/JAD-180847 31177210 PMC6697380

[B41] Falk E. B. Bassett D. S. (2017). Brain and social networks: fundamental building blocks of human experience. *Trends Cogn. Sci.* 21 674–690. 10.1016/j.tics.2017.06.009 28735708 PMC8590886

[B42] Fox N. C. Schott J. M. (2004). Imaging cerebral atrophy: normal ageing to Alzheimer’s disease. *Lancet* 363 392–394. 10.1016/S0140-6736(04)15441-X 15074306

[B43] Fratiglioni L. Wang H. X. (2007). Brain reserve hypothesis in dementia. *J. Alzheimers Dis.* 12 11–22. 10.3233/JAD-2007-12103 17851191

[B44] Freedman M. Binns M. A. Black S. E. Murphy C. Stuss D. T. (2013). Theory of mind and recognition of facial emotion in dementia: challenge to current concepts. *Alzheimer Dis. Assoc. Disord.* 27 56–61. 10.1097/WAD.0b013e31824ea5db 22407224

[B45] Frith C. D. Frith U. (2006). The neural basis of mentalizing. *Neuron* 50 531–534. 10.1016/j.neuron.2006.05.001 16701204

[B46] Fusar-Poli P. Placentino A. Carletti F. Landi P. Allen P. Surguladze S. (2009). Functional atlas of emotional faces processing: a voxel-based meta-analysis of 105 functional magnetic resonance imaging studies. *J. Psychiatry Neurosci.* 34, 418–432. 19949718 PMC2783433

[B47] Gallagher H. L. Frith C. D. (2003). Functional imaging of ‘theory of mind’. *Trends Cogn. Sci.* 7 77–83. 10.1016/S1364-6613(02)00025-6 12584026

[B48] García-Rodríguez B. Vincent C. Casares-Guillén C. Ellgring H. Frank A. (2012). The effects of different attentional demands in the identification of emotional facial expressions in Alzheimer’s disease. *Am. J. Alzheimers Dis. Demen.* 27 530–536. 10.1177/1533317512459797 22984090 PMC10697361

[B49] Greicius M. D. Srivastava G. Reiss A. L. Menon V. (2004). Default-mode network activity distinguishes Alzheimer’s disease from healthy aging: evidence from functional MRI. *Proc. Nat. Acad. Sci. USA* 101 4637–4642. 10.1073/pnas.0308627101 15070770 PMC384799

[B50] Habeck C. Risacher S. Lee G. J. Glymour M. M. Mormino E. Mukherjee S. (2012). Relationship between baseline brain metabolism measured using [^18^F]FDG PET and memory and executive function in prodromal and early Alzheimer’s disease. *Brain Imag. Behav.* 6 568–583. 10.1007/s11682-012-9208-x 23179062 PMC3532575

[B51] Heitz C. Noblet V. Phillipps C. Cretin B. Vogt N. Philippi N. (2016). Cognitive and affective theory of mind in dementia with Lewy bodies and Alzheimer’s disease. *Alzheimer’s Res. Therap.* 8:10. 10.1186/s13195-016-0179-9 26979460 PMC4793654

[B52] Hot P. Klein-Koerkamp Y. Borg C. Richard-Mornas A. Zsoldos I. Paignon Adeline A. (2013). Fear recognition impairment in early-stage Alzheimer’s disease: when focusing on the eyes region improves performance. *Brain Cogn.* 82 25–34. 10.1016/j.bandc.2013.02.001 23501701

[B53] Hughes C. Cassidy B. S. Faskowitz J. Avena-Koenigsberger A. Sporns O. Krendl A. C. (2019). Age differences in specific neural connections within the Default Mode Network underlie theory of mind. *Neuroimage* 191 269–277. 10.1016/j.neuroimage.2019.02.024 30794869 PMC6492272

[B54] Jack C. R. Jr. Bennett D. A. Blennow K. Carrillo M. C. Dunn B. Haeberlein S. B. (2018). NIA-AA Research Framework: Toward a biological definition of Alzheimer’s disease. *Alzheimers Demen.* 14 535–562. 10.1016/j.jalz.2018.02.018 29653606 PMC5958625

[B55] Jonker F. Wattjes M. Scherder E. (2017). Impaired behavioural self-awareness and affective Theory of Mind deficits following prefrontal cortex damage. *Neuropsychiatry* 7 750–758. 10.4172/Neuropsychiatry

[B56] Jorge Cardoso M. Leung K. Modat M. Keihaninejad S. Cash D. Barnes J. (2013). STEPS: Similarity and Truth Estimation for Propagated Segmentations and its application to hippocampal segmentation and brain parcelation. *Med. Image Anal.* 17 671–684. 10.1016/j.media.2013.02.006 23510558

[B57] Kalbe E. Schlegel M. Sack A. T. Nowak D. A. Dafotakis M. Bangard C. (2010). Dissociating cognitive from affective theory of mind: a TMS study. *Cortex* 46 769–780. 10.1016/j.cortex.2009.07.010 19709653

[B58] Kessels R. P. C. Waanders-OudeElferink M. van Tilborg I. (2021). Social cognition and social functioning in patients with amnestic mild cognitive impairment or Alzheimer’s dementia. *J. Neuropsy.* 15:12223. 10.1111/jnp.12223 32979297 PMC8247057

[B59] Krall S. C. Rottschy C. Oberwelland E. Bzdok D. Fox P. T. Eickhoff S. B. (2015). The role of the right temporoparietal junction in attention and social interaction as revealed by ALE meta-analysis. *Brain Struct. Funct.* 220 587–604. 10.1007/s00429-014-0803-z 24915964 PMC4791048

[B60] Kucyi A. Hodaie M. Davis K. D. (2012). Lateralization in intrinsic functional connectivity of the temporoparietal junction with salience- and attention-related brain networks. *J. Neurophys.* 108 3382–3392. 10.1152/jn.00674.2012 23019004

[B61] Kumfor F. Honan C. McDonald S. Hazelton J. L. Hodges J. R. Piguet O. (2017). Assessing the “social brain” in dementia: Applying TASIT-S. *Cortex* 93 166–177. 10.1016/j.cortex.2017.05.022 28662418

[B62] Kurth F. Zilles K. Fox P. T. Laird A. R. Eickhoff S. B. (2010). A link between the systems: functional differentiation and integration within the human insula revealed by meta-analysis. *Brain Struct. Funct.* 214 519–534. 10.1007/s00429-010-0255-z 20512376 PMC4801482

[B63] Laisney M. Bon L. Guiziou C. Daluzeau N. Eustache F. Desgranges B. (2013). Cognitive and affective Theory of Mind in mild to moderate Alzheimer’s disease. *J. Neuropsy.* 7 107–120. 10.1111/j.1748-6653.2012.02038.x 23088554

[B64] Lancaster J. L. Rainey L. H. Summerlin J. L. Freitas C. S. Fox P. T. Evans A. C. (1997). Automated labeling of the human brain: a preliminary report on the development and evaluation of a forward-transform method. *Human Brain Map.* 5 238–242. 10.1002/(SICI)1097-0193(1997)5:4<238::AID-HBM6>3.0.CO;2-4 PMC2860189 20408222

[B65] Lancaster J. L. Woldorff M. G. Parsons L. M. Liotti M. Freitas C. S. Rainey L. (2000). Automated Talairach atlas labels for functional brain mapping. *Human Brain Map.* 10 120–131. 10.1002/1097-0193(200007)10:3<120::AID-HBM30>3.0.CO;2-8 PMC6871915 10912591

[B66] Le Bouc R. Lenfant P. Delbeuck X. Ravasi L. Lebert F. Semah F. (2012). My belief or yours? Differential theory of mind deficits in frontotemporal dementia and Alzheimer’s disease. *Brain* 135(Pt 10), 3026–3038. 10.1093/brain/aws237 23065791

[B67] Leslie A. M. Friedman O. German T. P. (2004). Core mechanisms in “theory of mind”. *Trends Cogn. Sci.* 8 528–533. 10.1016/j.tics.2004.10.001 15556021

[B68] Li W. Mai X. Liu C. (2014). The default mode network and social understanding of others: what do brain connectivity studies tell us. *Front. Human Neurosci.* 8:74. 10.3389/fnhum.2014.00074 24605094 PMC3932552

[B69] Lieberman M. D. (2007). Social cognitive neuroscience: a review of core processes. *Ann. Rev. Psychol*. 58 259–289. 10.1146/annurev.psych.58.110405.085654 17002553

[B70] Lucena A. T. Bhalla R. K. Belfort Almeida, Dos Santos T. T. Dourado M. C. N. (2020). The relationship between theory of mind and cognition in Alzheimer’s disease: A systematic review. *J. Clin. Exp. Neuropsychol.* 42 223–239. 10.1080/13803395.2019.1710112 31902277

[B71] Mars R. B. Neubert F.-X. Noonan M. P. Sallet J. Toni I. Rushworth M. F. S. (2012). On the relationship between the “default mode network” and the “social brain”. *Front. Human Neurosci.* 6:189–189. 10.3389/fnhum.2012.00189 22737119 PMC3380415

[B72] Martinez M. Multani N. Anor C. J. Misquitta K. Tang-Wai D. F. Keren R. (2018). Emotion detection deficits and decreased empathy in patients with Alzheimer’s disease and parkinson’s disease affect caregiver mood and burden. *Front. Aging Neurosci.* 10:120. 10.3389/fnagi.2018.00120 29740312 PMC5928197

[B73] McKhann G. M. Knopman D. S. Chertkow H. Hyman B. T. Jack C. R. Kawas C. H. (2011). The diagnosis of dementia due to Alzheimer’s disease: Recommendations from the National Institute on Aging-Alzheimer’s Association workgroups on diagnostic guidelines for Alzheimer’s disease. *Alzheimer’s Demen.* 7 263–269. 10.1016/j.jalz.2011.03.005 21514250 PMC3312024

[B74] Mier D. Lis S. Neuthe K. Sauer C. Esslinger C. Gallhofer B. (2010). The involvement of emotion recognition in affective theory of mind. *Psychophysiology* 47 1028–1039. 10.1111/j.1469-8986.2010.01031.x 20456660

[B75] Miguel F. K. Caramanico R. B. Huss E. Y. Zuanazzi A. C. (2017). Validity of the reading the mind in the eyes test in a brazilian sample. *Paidéia (Ribeirão Preto)* 27 16–23. 10.1590/1982-43272766201703

[B76] Miller C. A. (2006). Developmental relationships between language and theory of mind. *Am. J. Speech-Lang. Pathol.* 15 142–154. 10.1044/1058-0360(2006/014) 16782686

[B77] Mitchell R. L. Phillips L. H. (2015). The overlapping relationship between emotion perception and theory of mind. *Neuropsychologia* 70 1–10. 10.1016/j.neuropsychologia.2015.02.018 25687032

[B78] Moreau N. Rauzy S. Viallet F. Champagne-Lavau M. (2016). Theory of mind in Alzheimer disease: Evidence of authentic impairment during social interaction. *Neuropsychology* 30 312–321. 10.1037/neu0000220 26146852

[B79] Otti A. Wohlschlaeger A. M. Noll-Hussong M. (2015). Is the Medial Prefrontal Cortex Necessary for Theory of Mind? *PLoS One* 10:e0135912–e0135912. 10.1371/journal.pone.0135912 26301900 PMC4547759

[B80] Peelle J. E. Cusack R. Henson R. N. A. (2012). Adjusting for global effects in voxel-based morphometry: Gray matter decline in normal aging. *Neuroimage* 60 1503–1516. 10.1016/j.neuroimage.2011.12.086 22261375 PMC3898996

[B81] Perner J. Aichhorn M. Kronbichler M. Staffen W. Ladurner G. (2006). Thinking of mental and other representations: The roles of left and right temporo-parietal junction. *Soc. Neurosci.* 1 245–258. 10.1080/17470910600989896 18633791

[B82] Petersen R. C. (2004). Mild cognitive impairment as a diagnostic entity. *J. Inter. Med.* 256 183–194. 10.1111/j.1365-2796.2004.01388.x 15324362

[B83] Phillips L. H. Scott C. Henry J. D. Mowat D. Bell J. S. (2010). Emotion perception in Alzheimer’s disease and mood disorder in old age. *Psychol. Aging* 25 38–47. 10.1037/a0017369 20230126

[B84] Poletti M. Enrici I. Adenzato M. (2012). Cognitive and affective Theory of Mind in neurodegenerative diseases: neuropsychological, neuroanatomical and neurochemical levels. *Neurosci. Biobehav. Rev.* 36 2147–2164. 10.1016/j.neubiorev.2012.07.004 22819986

[B85] Postema M. C. De Marco M. Colato Venneri A. (2019). A study of within-subject reliability of the brain’s default-mode network. *Magn. Reson. Mater. Phy.* 32 391–405. 10.1007/s10334-018-00732-0 30730023 PMC6525123

[B86] Ramanan S. de Souza L. C. Moreau N. Sarazin M. Teixeira A. L. Allen Z. (2017). Determinants of theory of mind performance in Alzheimer’s disease: A data-mining study. *Cortex* 88 8–18. 10.1016/j.cortex.2016.11.014 28012370

[B87] Rowe A. D. Bullock P. R. Polkey C. E. Morris R. G. (2001). “Theory of mind” impairments and their relationship to executive functioning following frontal lobe excisions. *Brain* 124(Pt 3) 600–616. 10.1093/brain/124.3.600 11222459

[B88] Saltzman J. Strauss E. Hunter M. Archibald S. (2000). Theory of mind and executive functions in normal human aging and Parkinson’s disease. *J. Int. Neuropsychol. Soc.* 6 781–788. 10.1017/s1355617700677056 11105468

[B89] Samson D. Apperly I. A. Chiavarino C. Humphreys G. W. (2004). Left temporoparietal junction is necessary for representing someone else’s belief. *Nat. Neurosci.* 7 499–500. 10.1038/nn1223 15077111

[B90] Sapey-Triomphe L.-A. Heckemann R. A. Boublay N. Dorey J.-M. Hénaff M.-A. Rouch I. (2015). Neuroanatomical correlates of recognizing face expressions in mild stages of Alzheimer’s disease. *PLoS One* 10:e0143586–e0143586. 10.1371/journal.pone.0143586 26673928 PMC4684414

[B91] Saxe R. Kanwisher N. (2003). People thinking about thinking people. The role of the temporo-parietal junction in “theory of mind”. *Neuroimage* 19 1835–1842. 10.1016/s1053-8119(03)00230-1 12948738

[B92] Saxe R. Wexler A. (2005). Making sense of another mind: the role of the right temporo-parietal junction. *Neuropsychologia* 43 1391–1399. 10.1016/j.neuropsychologia.2005.02.013 15936784

[B93] Schilbach L. Eickhoff S. B. Rotarska-Jagiela A. Fink G. R. Vogeley K. (2008). Minds at rest? Social cognition as the default mode of cognizing and its putative relationship to the “default system” of the brain. *Consciou. Cogn.* 17 457–467. 10.1016/j.concog.2008.03.013 18434197

[B94] Schlaffke L. Lissek S. Lenz M. Juckel G. Schultz T. Tegenthoff M. (2015). Shared and nonshared neural networks of cognitive and affective theory-of-mind: a neuroimaging study using cartoon picture stories. *Human Brain Map.* 36 29–39. 10.1002/hbm.22610 25131828 PMC6869702

[B95] Schmahmann J. D. (2019). The cerebellum and cognition. *Neurosci. Lett.* 688 62–75. 10.1016/j.neulet.2018.07.005 29997061

[B96] Schurz M. Radua J. Aichhorn M. Richlan F. Perner J. (2014). Fractionating theory of mind: a meta-analysis of functional brain imaging studies. *Neurosci. Biobehav. Rev.* 42 9–34. 10.1016/j.neubiorev.2014.01.009 24486722

[B97] Serafin M. Surian F. (2004). Il Test degli Occhi: uno strumento per valutare la “teoria della mente”. *Giornale Italiano di Psicologia* 4 839–860. 10.1421/18849

[B98] Synn A. Mothakunnel A. Kumfor F. Chen Y. Piguet O. Hodges J. R. (2018). Mental states in moving shapes: Distinct cortical and subcortical contributions to Theory of Mind impairments in dementia. *J. Alzheimers Dis.* 61 521–535. 10.3233/jad-170809 29172002

[B99] Takenoshita S. Hayashi S. Shinya T. Miki T. Yokota O. Maki Y. (2020). Sally–Anne test and regional cerebral blood flow in Alzheimer’s disease dementia. *Psychogeriatrics* 20 549–556. 10.1111/psyg.12533 32153079

[B100] Takenoshita S. Terada S. Yokota O. Kutoku Y. Wakutani Y. Nakashima M. (2018). Sally-Anne test in patients with Alzheimer’s disease dementia. *J. Alzheimers Dis.* 61 1029–1036. 10.3233/jad-170621 29332047

[B101] Torres B. Santos R. L. Sousa M. F. Simões Neto J. P. Nogueira M. M. Belfort T. T. (2015). Facial expression recognition in Alzheimer’s disease: a longitudinal study. *Arquivos de Neuropsiquiatria* 73 383–389. 10.1590/0004-282x20150009 26017202

[B102] Torres M. De Melo Fádel B. Santos De Carvalho R. L. Belfort A. Dos Santos T. T. (2019). Facial expression recognition in Alzheimer’s disease: A systematic review. *J. Clin. Exp. Neuropsy.* 41 192–203. 10.1080/13803395.2018.1501001 30088784

[B103] Valera-Bermejo J. M. De Marco M. Mitolo M. McGeown W. J. Venneri A. (2020). Neuroanatomical and cognitive correlates of domain-specific anosognosia in early Alzheimer’s disease. *Cortex* 129 236–246. 10.1016/j.cortex.2020.04.026 32534349

[B104] Valle A. Massaro D. Castelli I. Marchetti A. (2015). Theory of Mind development in adolescence and early adulthood: The growing complexity of recursive thinking ability. *Eur. J. Psychol.* 11 112–124. 10.5964/ejop.v11i1.829 27247645 PMC4873097

[B105] Van Overwalle F. Baetens K. Mariën P. Vandekerckhove M. (2014). Social cognition and the cerebellum: a meta-analysis of over 350 fMRI studies. *Neuroimage* 86 554–572. 10.1016/j.neuroimage.2013.09.033 24076206

[B106] Vellante M. Baron-Cohen S. Melis M. Marrone M. Petretto D. R. Masala C. (2013). The “Reading the Mind in the Eyes” test: Systematic review of psychometric properties and a validation study in Italy. *Cogn. Neuropsy.* 18 326–354. 10.1080/13546805.2012.721728 23106125 PMC6345369

[B107] Wade M. Prime H. Jenkins J. M. Yeates K. O. Williams T. Lee K. (2018). On the relation between theory of mind and executive functioning: A developmental cognitive neuroscience perspective. *Psychon. Bull. Rev.* 25 2119–2140. 10.3758/s13423-018-1459-0 29619648

[B108] Wakefield S. J. McGeown W. J. Shanks M. F. Venneri A. (2014). Differentiating normal from pathological brain ageing using standard neuropsychological tests. *Curr. Alzheimer Res.* 11 765–772. 10.2174/156720501108140910121631 25212915

[B109] Wang Y. Li T.-Q. (2015). Dimensionality of ICA in resting-state fMRI investigated by feature optimized classification of independent components with SVM. *Front. Human Neurosci.* 9:259–259. 10.3389/fnhum.2015.00259 26005413 PMC4424860

[B110] Woo B. K. Harwood D. G. Melrose R. J. Mandelkern M. A. Campa O. M. Walston A. (2010). Executive deficits and regional brain metabolism in Alzheimer’s disease. *Int. J. Geriat. Psychiatry* 25 1150–1158. 10.1002/gps.2452 20069587

[B111] Yildirim E. Soncu Buyukiscan E. Demirtas-Tatlidede A. Bilgiç B. Gurvit H. (2020). An investigation of affective theory of mind ability and its relation to neuropsychological functions in Alzheimer’s disease. *J. Neuropsychol.* 14 399–415. 10.1111/jnp.12207 32212244

[B112] Zaitchik D. Koff E. Brownell H. Winner E. Albert M. (2004). Inference of mental states in patients with Alzheimer’s disease. *Cogn. Neuropsy.* 9 301–313. 10.1080/13546800344000246 16571588

[B113] Zhang Z. Liu Y. Jiang T. Zhou B. An N. Dai H. (2012). Altered spontaneous activity in Alzheimer’s disease and mild cognitive impairment revealed by Regional Homogeneity. *Neuroimage* 59 1429–1440. 10.1016/j.neuroimage.2011.08.049 21907292

