# Supplementary material for: Large-Scale Functional Networks, Cognition and Brain Structures Supporting Social Cognition and Theory of Mind Performance in Prodromal to Mild Alzheimer’s Disease
Source: Front Aging Neurosci. 2021 Nov 17;13:766703. doi: 10.3389/fnagi.2021.766703 (PMC8636093; doi:10.3389/fnagi.2021.766703)
Supplement: Supplementary file 1 [file Data_Sheet_1.docx]

Neural correlates of Theory of Mind in early Alzheimer’s disease

**Jose Manuel Valera-Bermejo 1, Matteo De Marco 2, Micaela Mitolo 3, Chiara Cerami 4,5, Alessandra Dodich 6, Annalena Venneri 1,2*.**

^1^ Department of Neuroscience, University of Sheffield, Sheffield, UK;

^2^ Brunel University London, London, UK

^3^ IRCCS Istituto delle Scienze Neurologiche di Bologna, Functional and Molecular Neuroimaging Unit, Bologna, Italy;

^4^ Scuola Universitaria di Studi Superiori IUSS Pavia, Pavia, Italy

^5^ Cognitive Computational Neuroscience Research Unit, IRCCS Mondino Foundation, Pavia, Italy

^6^ CIMeC Università degli Studi di Trento, Rovereto, Italy

***Supplementary Material***

In order to characterise the neurological status of patients in more detail, we compared their neurofunctional architecture with that of the healthy control group. This was carried out with the same rationale as the ‘patients-vs.-controls’ grey-matter map comparison described in the main manuscript.

A resting-state fMRI sequence was also acquired for each of the 34 controls. This was based on the same technical specifications as those of the patients’ images. Similarly, the same routines were applied to preprocessing of these images.

A second group-level independent component analysis (ICA) was thus run, this time across the entire cohort of patients and healthy controls (*n* = 80). This was carried out using the GIFT toolbox via the same methodology described in the manuscript that was applied to the first ICA.

The output of this second ICA was reviewed to identify and select the five components corresponding to the five networks of interest investigated in the main section of the study. An independent-sample *t*-test model was then run to compare patients and healthy controls for each network, controlling for age and years of education. A conservative cluster-forming threshold of *p* < 0.005 was applied to these models and only clusters surviving a Family-Wise Error-corrected *p* < 0.05 were reported as significant.

Two clusters emerged as significant from this set of analyses. A first difference was found in the posterior default-mode network: this pathway was more strongly expressed among healthy controls in postero-medial regions corresponding to Brodmann area 7 and 31. This is typically considered the core of the posterior default-mode network, and this finding replicates well-established evidence of regional network down-regulation observed in patients with mild cognitive impairment and in patients with dementia of the Alzheimer’s type (Badhwar et al., 2017; Balthazar et al., 2014; Garcés et al., 2014; Greicius et al., 2004; Jin et al., 2012; Sarli et al., 2021). The second significant cluster emerged from the analysis of the right frontoparietal network. Patients showed stronger network expression in a midline cluster similarly centred in the precuneus, but extending between the parietal and occipital lobes (Brodmann areas 7 and 19). The right frontoparietal network, typically expressed in the right dorsolateral prefrontal and right posterior parietal cortices, is not selectively targeted by the neuropathological processes of Alzheimer’s disease (as is the case for the default-mode network). Nevertheless, the expression of this network in the precuneus is one of the pathways showing the clearest longitudinal degradation in patients with Alzheimer’s disease (Hafkemeijer et al., 2017). As our patients were all in the early-stage of the disease, it is possible that this specific territory is subjected to functional alterations during the earlies disease stages in the attempt to compensate for the ongoing degradation processes and this results in a temporary, short-lived increase in functional connectivity, destined to show, instead, an inverse trend during the later stages of disease. All findings are illustrated in **Supplementary Figure 1** and described in **Supplementary Table 1**.

Furthermore, we also investigated group differences in the salience network at a more liberal and exploratory threshold equal to a cluster-forming *p* < 0.05. We did so to identify possible trends indicating up-regulations in this pathway as often found in patients at the early stage of Alzheimer’s disease (Badhwar et al., 2017). Patients showed a trend towards stronger expression of the salience network in a set of regions including the right insula and the posterior cingulate cortex (**Supplementary Figure 2**). This trend suggests hyperconnectivity within the salience network as well as network encroachment in the form of an increased cross-talk between the salience network and the default-mode network, of which the posterior cingulate cortex is the core hub.

**References**

Badhwar, A. Tam, A., Dansereau, C., Orban, P., Hoffstaedter, F. & Bellec, P. (2017). Resting-state network dysfunction in Alzheimer’s disease: a systematic review and meta-analysis. Alzheimers Dement. (Amst.) 8, 73–85. doi: 10.1016/j.dadm.2017.03.007

Balthazar, M. L. F., de Campos, B. M., Franco, A. R., Damasceno, B. P. & Cendes, F. (2014). Whole cortical and default mode network mean functional connectivity as potential biomarkers for mild Alzheimer’s disease. Psychiatry Res. 221, 37–42. doi: 10.1016/j.pscychresns.2013.10.010

Garcés, P., Pineda-Pardo, J. A., Canuet, L., Aurtenetxe, S., López, M. E., Marcos, A., et al. (2014). The default mode network is functionally and structurally disrupted in amnestic mild cognitive impairment - A bimodal MEG-DTI study. Neuroimage Clin. 6, 214–221. doi: 10.1016/j.nicl.2014.09.004

Greicius, M. D., Srivastava, G., Reiss, A. L. & Menon, V. (2004). Default-mode network activity distinguishes Alzheimer’s disease from healthy aging: evidence from functional MRI. Proc. Natl. Acad. Sci. USA 101, 4637–4642. doi: 10.1073/pnas.0308627101

Hafkemeijer, A., Möller, C., Dopper, E. G. P. Jiskoot, L. C., van den Berg-Huysmans, A. A., van Swieten, J. C., et al. (2017). A longitudinal study on resting state functional connectivity in behavioral variant frontotemporal dementia and Alzheimer's disease. J. Alzheimers Dis. 55, 521-537. doi: 10.3233/jad-150695.

Jin, M. W., Pelak, V. S. & Cordes, D. (2012). Aberrant default mode network in subjects with amnestic mild cognitive impairment using resting-state functional MRI. Magn. Reson. Imaging 30, 48–61. doi: 10.1016/j.mri.2011.07.007

Sarli, G., De Marco, M., Hallikainen, M., Soininen, H., Bruno, G. & Venneri, A. (2021). Regional strength of large-scale functional brain networks is associated with regional volumes in older adults and in Alzheimer’s disease. Brain Connect. 11, 201-2012. doi: 10.1089/brain.2020.0899

**Supplementary Table 1**

| **Brain Region** | **Hemisphere** | **Brodmann Area** | **Z Score at Local Maximum** | **Cluster-Level pFWE** | **Cluster Extent (voxels)** | **Talairach Coordinates** | | |
| --- | --- | --- | --- | --- | --- | --- | --- | --- |
|  |  |  |  |  |  | **x** | **y** | **z** |
| *Posterior Default-Mode Network - Controls > Patients* | | | | | | | | |
| Precuneus | R | 7 | 3.37 | 0.032 | 253 | 2 | -74 | 39 |
| Precuneus | R | 7 | 3.34 |  |  | 4 | -59 | 31 |
| Cuneus | L | 7 | 3.22 |  |  | -2 | -72 | 31 |
| Precuneus | R | 31 | 3.21 |  |  | 14 | -65 | 25 |
| Precuneus | R | 31 | 3.02 |  |  | 8 | -70 | 35 |
| Cuneus | R | 7 | 3 |  |  | 12 | -70 | 33 |
| Precuneus | R | 31 | 2.93 |  |  | 2 | -63 | 27 |
| *Right Frontoparietal Network - Patients > Controls* | | | | | | | | |
| Precuneus | L | 31 | 4.09 | 0.005 | 344 | -2 | -70 | 29 |
| Cuneus | L | 19 | 3.46 |  |  | -2 | -82 | 37 |
| Cuneus | L | 19 | 3.37 |  |  | -2 | -78 | 32 |
| Precuneus | R | 7 | 3.22 |  |  | 16 | -68 | 42 |

**Supplementary Figure 1**

**
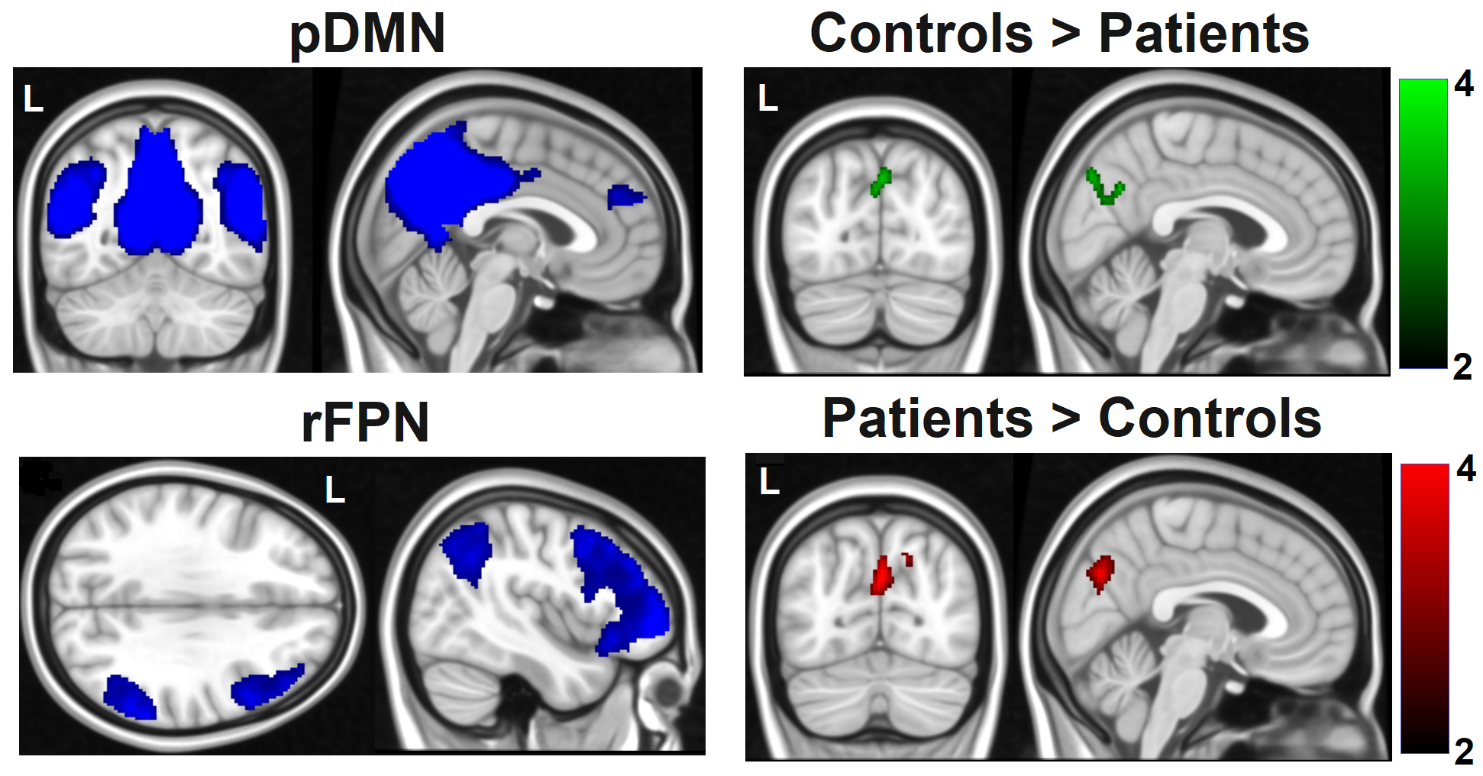
**

Differences (significant at a cluster-forming threshold equal to *p* < 0.005) between patients and healthy controls in two of the five large-scale pathways investigated in this study. Network contours (obtained via one-sample *t*-tests) are shown on the left hand-side. The strength of the statistical effect is illustrated on the right hand-side. pDMN: posterior default-mode network; rFPM: right frontoparietal network.

**Supplementary Figure 2**


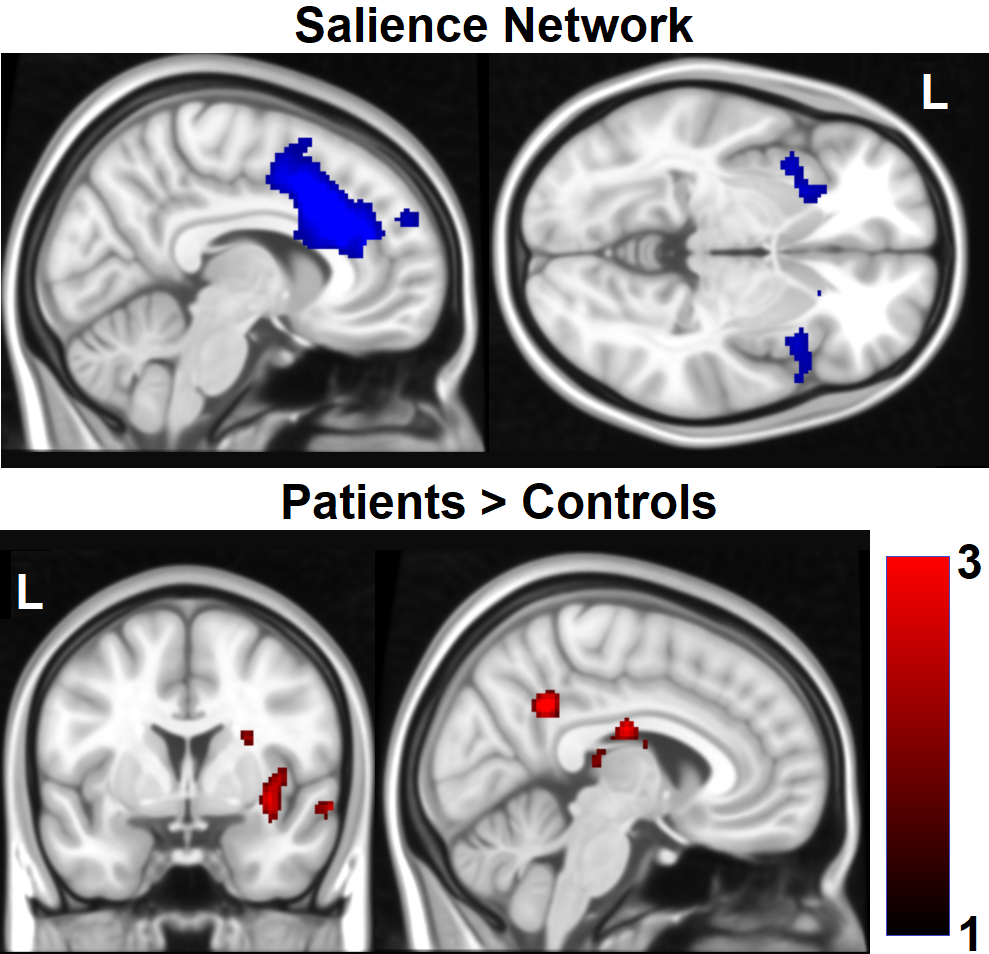


Differences (significant at a liberal and exploratory cluster-forming threshold equal to *p* < 0.05) between patients and controls in the salience network. Network contours (obtained via a one-sample *t*-test) are coloured in blue and shown above. The map of the statistical effect is illustrated below.
